# Probiotic activities of *Rhizobium laguerreae* on growth and quality of spinach

**DOI:** 10.1038/s41598-017-18632-z

**Published:** 2018-01-10

**Authors:** Alejandro Jiménez-Gómez, José David Flores-Félix, Paula García-Fraile, Pedro F. Mateos, Esther Menéndez, Encarna Velázquez, Raúl Rivas

**Affiliations:** 1 Microbiology and Genetics Department, University of Salamanca, 37007 Salamanca, Spain; 2 Spanish-Portuguese Institute for Agricultural Research (CIALE), Villamayor, Salamanca, Spain; 3 Institute of Microbiology ASCR,v.v.i., Vídeňská 1083, 142 20 Prague, Czech Republic; 4 Associated R&D Unit, USAL-CSIC (IRNASA), Salamanca, Spain; 5 Present Address: ICAAM - Instituto de Ciências Agrárias e Ambientais Mediterrânicas, Universidade de Évora, Pólo da Mitra, Ap. 94, 7002-554 Évora, Portugal

## Abstract

The growing interest in a healthy lifestyle and in environmental protection is changing habits regarding food consumption and agricultural practices. Good agricultural practice is indispensable, particularly for raw vegetables, and can include the use of plant probiotic bacteria for the purpose of biofertilization. In this work we analysed the probiotic potential of the rhizobial strain PEPV40, identified as *Rhizobium laguerreae* through the analysis of the *recA* and *atpD* genes, on the growth of spinach plants. This strain presents several *in vitro* plant growth promotion mechanisms, such as phosphate solubilisation and the production of indole acetic acid and siderophores. The strain PEPV40 produces cellulose and forms biofilms on abiotic surfaces. GFP labelling of this strain showed that PEPV40 colonizes the roots of spinach plants, forming microcolonies typical of biofilm initiation. Inoculation with this strain significantly increases several vegetative parameters such as leaf number, size and weight, as well as chlorophyll and nitrogen contents. Therefore, our findings indicate, for the first time, that *Rhizobium laguerreae* is an excellent plant probiotic, which increases the yield and quality of spinach, a vegetable that is increasingly being consumed raw worldwide.

## Introduction

The consumption of salads, particularly those sold as ready-to-eat that include the leaves of different vegetables, is increasing worldwide^1^, but so too is the number of outbreaks of bacterial foodborne illnesses caused by eating raw leaves^2,3^. The pathogenic bacteria associated with fresh vegetables may come from irrigation water and from the manure or compost used to fertilize crops. This means that on-farm food safety practices are essential for preventing the microbial hazards linked to the consumption of raw food products^4^. These safety practices have been defined taking into account not only health safety and food quality, but also the environmental sustainability of agriculture^4^. One of the main challenges faced by the those establishing agricultural practices is to protect the environment by replacing chemical fertilizers by biofertilizers^5,6^. Biofertilizers include plant probiotic bacteria (PPB)^7^, which have several direct and indirect *in vitro* plant growth promotion (PGP) mechanisms, such as nitrogen fixation, phosphate solubilisation and the production of different compounds like phytohormones or siderophores^8^. We have recently shown that probiotic *Rhizobium* strains produce cellulose and biofilms involved in root colonization^9^, which is an important step for promoting plant growth^8^.

Many rhizospheric bacteria have several PGP mechanisms and act as potential candidates for use as a biofertilizer^10^; however, the rhizosphere is also a source of pathogens^11,12^. Therefore, food safety practices require the use of innocuous bacteria in biofertilization, particularly on vegetables consumed raw^13^. Rhizobia is a group of rhizospheric bacteria that establish nitrogen-fixing symbiosis with legumes, and so far no public or environmental health problems have been reported regarding their use as biofertilizers^13,14^. In addition to symbiotic nitrogen fixation, rhizobia have other PGP mechanisms, such as phosphate solubilisation and the production of siderophores and the phytohormone indole acetic acid (IAA)^9,13,15–22^. This suggests that these bacteria can also be used as a plant probiotic for non-legumes, a situation that has recently been reported for some cereals, fruits, and vegetables^9,13,15,16,19–21,23–26^.

It is well known that the inclusion of fruits and vegetables in the human diet is inversely related to mortality in cancer patients^27^, as well as decreasing the risk of cardiovascular diseases^28^ and other chronic complaints such as obesity and type 2 diabetes mellitus^29^. Among the many diets currently being studied, the Mediterranean diet, which includes the high consumption of fresh vegetables and fruits, is associated with a lower risk of cancer mortality, and a lower rate of several types of cancer^30,31^, cardiovascular diseases^28^, other metabolic and chronic complaints^29,32,33^, cognitive decline^30^, and probably Alzheimer’s disease^34^.

Spinach (*Spinacia oleracea* L.), one of the vegetables included in the Mediterranean diet, stands out for its nutritional value, as it contains vitamins, amino acids, and minerals^35^. It is currently one of the most widely grown vegetables, with more than 23 million tons produced worldwide in 2013^36^. Although spinach is traditionally consumed cooked, it can also be eaten raw in salads, including those sold ready-to-eat, which commonly contain spinach leaves that are smaller and more tender than those used for cooking. Therefore, owing to its popularity and raw consumption, spinach could be considered a candidate for biofertilization with plant probiotic bacteria. Although the effect on the growth of spinach after being inoculated with some strains of *Bacillus*, *Paenibacillus*, and *Pseudomonas* of has been analyzed^37^, no data are available on the effects of *Rhizobium* species on its growth and quality.

As previously noted, rhizobia include several genera and species, of which some soybean-nodulating strains belonging to the genus *Bradyrhizobium* are the most widely used inoculants^38^, although species of *Mesorhizobium*, *Ensifer* (*Sinorhizobium*), and *Rhizobium* are used as inoculants for other legumes^39^. Of the aforementioned genera, *Rhizobium* is the most widely studied for its potential use as a probiotic of non-legumes. This is particularly the case for *R. leguminosarum* ^13^, since this species is found worldwide in the nodules of several legumes^40^. Nevertheless, the effect of other more recently described species, such as *Rhizobium laguerreae* also found worldwide^41^, on the growth of non-legumes remains unknown. The aim of this study was therefore to analyse the interaction between *Rhizobium laguerreae* and spinach plants, starting from the first steps of root colonization through to the final yield of leaves at different stages of growth.

## Results

### Phylogenetic analysis

**Figure 1:**
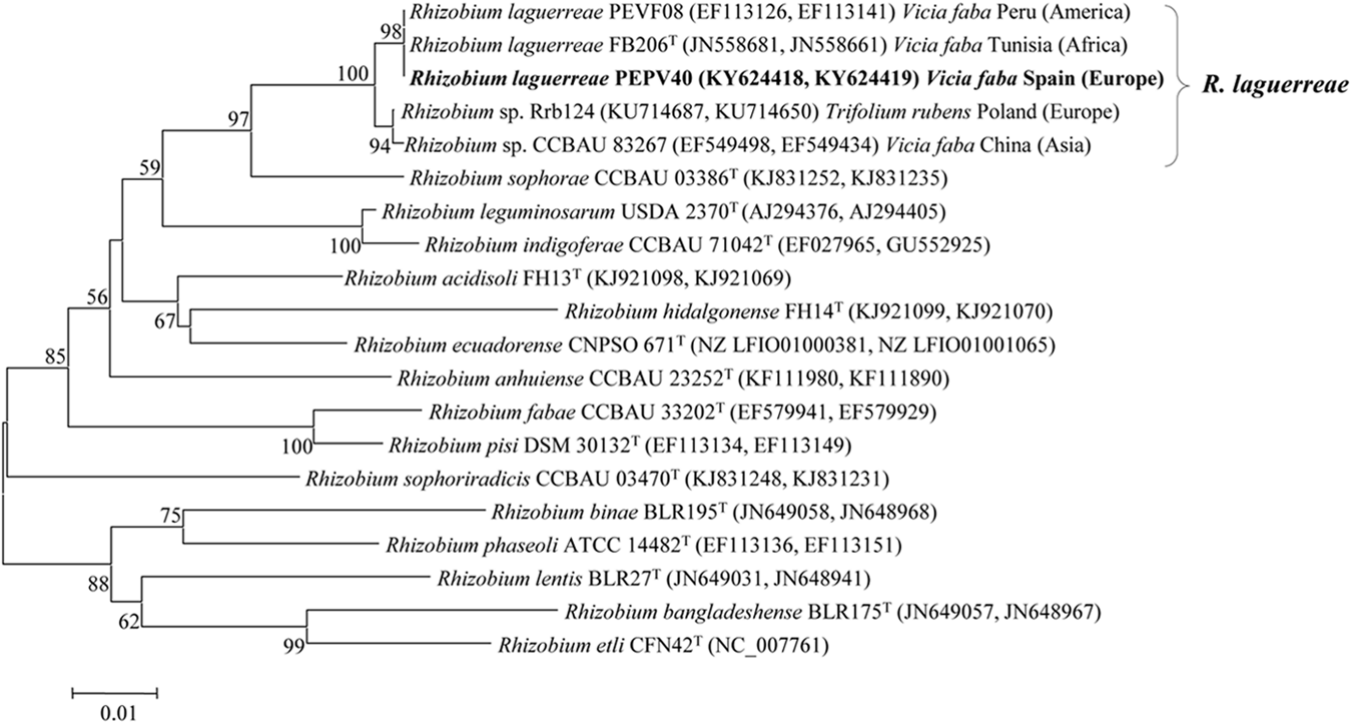
Phylogenetic analysis. The tree based on partial concatenated sequences of *recA* and *atpD* genes (by this order and with a total of 760 positions) showed the phylogenetic position of strain PEPV40 within genus *Rhizobium*. The significance of each branch is indicated by a bootstrap value (in percentage) calculated for 1000 subsets (only values higher than 50% are indicated). Bar, 1 substitutions per 100 nucleotide positions.

The strain PEPV40 was identified using two housekeeping genes, *recA* and *atpD*, present in almost all *Rhizobium* species. The results revealed a 100% match with the type strain of the species *Rhizobium laguerreae*, isolated from *Vicia faba* nodules in Tunisia. The analysis of the concatenated *recA* and *atpD* genes showed that PEPV40 was also closely related (similarity values higher than 99%) to strains isolated in several continents, such as PEVF08, isolated from *Vicia faba* in Peru, BIHB 1107 isolated from *Pisum sativum* nodules in India, and Rrb124 isolated in Poland (Fig. 1). These results show that *R. laguerreae* strains are present in soils from different continents, thereby permitting the global use of this species as a plant probiotic.

### *In vitro* PGP traits

The strain PEPV40 produced 56.1 mg l^−1^ of indole acetic acid when grown in JMM liquid medium supplemented with tryptophan; however, the concentration of IAA was lower (0.14 mg l^−1^) when measured by HPLC. PEPV40 also grew on chrome azurol S (CAS) indicator medium, where the colonies were surrounded by a yellow-orange halo (6 mm radius around colonies), indicating siderophore production. In medium O-CAS, the strain produced a colour change from greenish-blue to clear light yellow, which suggested that PEPV40 could produce carboxylate-type siderophores as shown by Pérez-Miranda *et al*.^42^. In addition, PEPV40 solubilized low amounts of phosphate, forming clear halos around their colonies on Pikovskaya’s agar (1.5 mm radius around colonies), which were not detected when tricalcium or hydroxyapatite were used as the P source. These results show that the strain PEPV40 has several direct and indirect mechanisms of plant growth promotion *in vitro*.

### Biofilm and cellulose production

**Figure 2:**
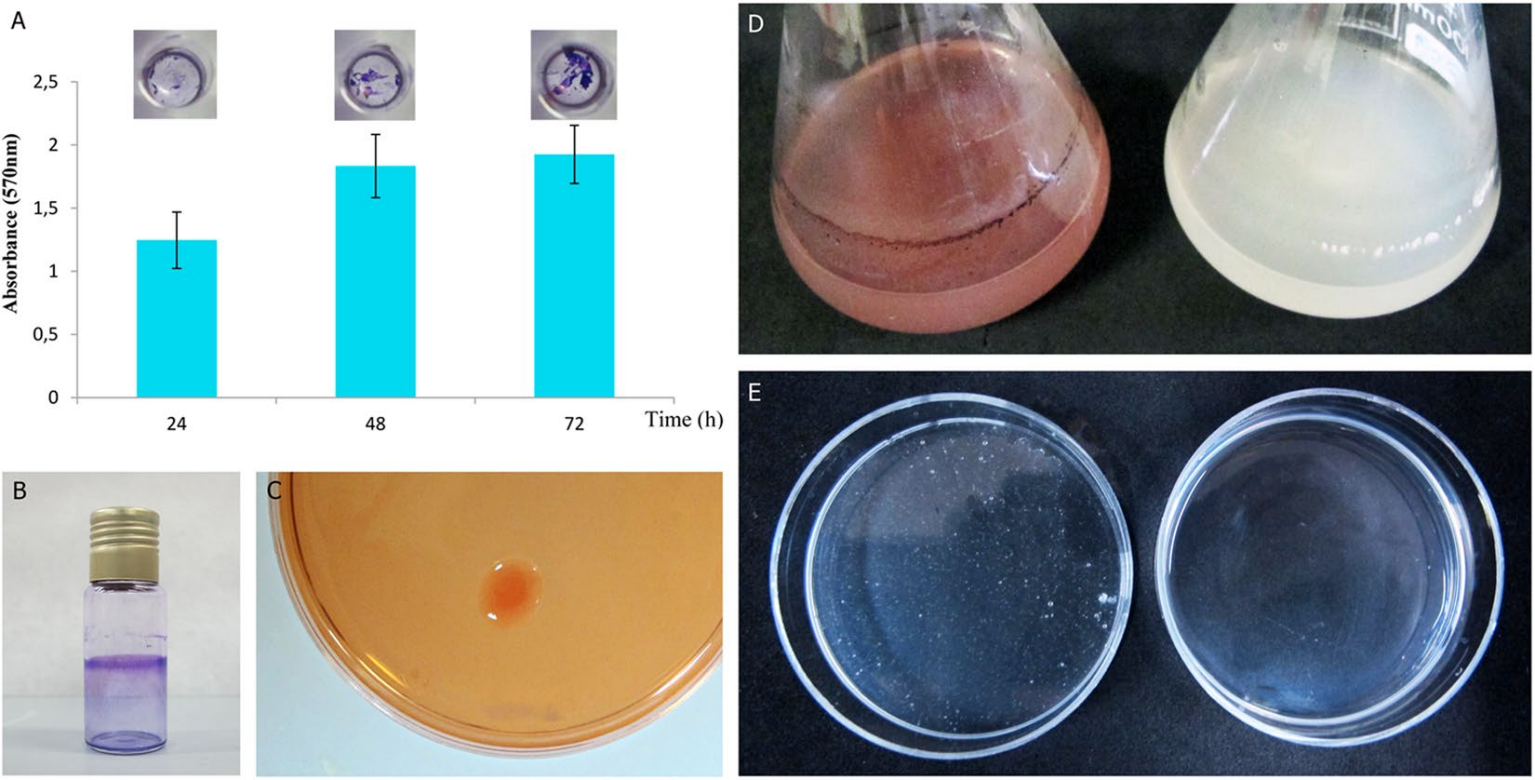
Biofilms and cellulose formation. Attachment of strain PEPV40 to abiotic surfaces observed in polystyrene microtiter plates along the time (Bars indicate the standard error. The experiment was repeated three times) (**A**) and in borosilicate glass tubes (**B**). Cellulose formed by the strain in plates containing Congo Red (**C**) and in YMB liquid medium with Congo Red (**D**, left) and without (**D**, right). Flocs formed by the strain in YMB liquid medium (**E**, left) and after 2 h incubation at 37 °C in pH 5 PCA buffer containing 10 U/ml *Trichoderma viride* commercial cellulase (**E**, right).

Strain PEPV40 produced biofilms on abiotic surfaces, with an increase in the amounts of crystal violet retained by the cells attached to the micro-wells over time (Fig. 2A). PEPV40 cells also attached to the walls of borosilicate tubes (Fig. 2B). Furthermore, it was observed that PEPV40 colonies were red after seven days of incubation on plates containing Congo Red. The intensity of the colour was comparable to that recorded for other *Rhizobium* strains^43^, which indicated that PEPV40 could produce a moderate amount of a polysaccharide with 1-4β glycosidic bonds. Cellulose was the most likely candidate, as the flocs formed by this strain were dissolved after incubation with commercial cellulases (Fig. 2C,D and E). The ability of PEPV40 to produce biofilms together with several molecules involved in plant growth development suggests that this strain could act as a plant growth promoter. However, complementary assay experiments are needed to confirm this.

### Colonization of spinach roots and *in vitro* growth promotion of seedlings

**Figure 3:**
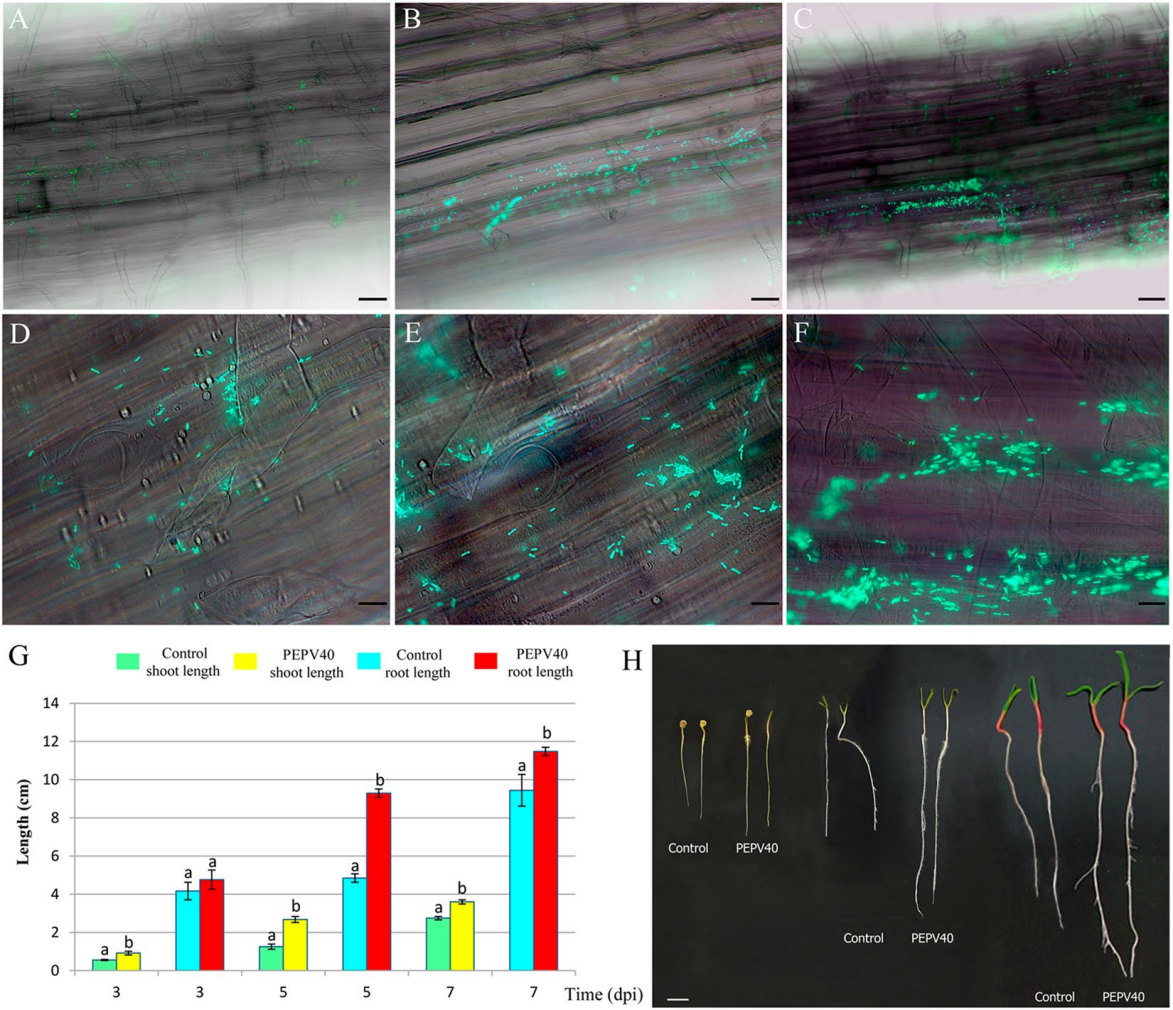
Bacterial root colonization. Fluorescence optical micrographs of spinach seedlings roots obtained at 3 (**A**,**D**), 5 (**B**,**E**) and 7 (**C**,**F**) days after inoculation with GFP-tagged cells of strain PEPV40 (**A**,**B**, and **C**, bar 60 µm, and **D**,**E** and **F**, bar 12 µm). The micrographs show the ability of strain PEPV40 to colonize the roots surfaces of spinach and the initiation of microcolonies. Effect of the strain PEPV40 inoculation in the early steps of spinach seedlings: Evolution of shoot and root length (**G**). Bars indicate the standard error. Histogram bars marked with the same letter in each treatment are not significantly different from each other at *P* = 0.05 according to Fisher’s Protected LSD (Least Significant Differences). Uninoculated and inoculated spinach seedlings from the *in vitro* experiment (H, bar 1 cm).

It was observed by fluorescence microscopy that GFP-tagged PEPV40 attached to the root surfaces of spinach seedlings inoculated with the strain that increased over time (Fig. 3A to F), revealing the typical microcolonies of biofilm initiation (Fig. 3D,F). In addition, significant increases were observed in both the shoot and root length of spinach seedlings after three days of inoculation with PEPV40. Moreover, this increase in length continued to reach over 90% on day 5 and over 20% on day 7 (Fig. 3G,H, Table 1). These results confirm the presence of positives effects on spinach plants at the early stages of development after being inoculated with PEPV40, suggesting that this strain may have probiotic potential, at least on spinach.

**Table 1:** Results from *in vitro* growth promotion experiment.

Treatment	Shoot length (±S.E.)* (cm)	Root length (±S.E.)* (cm)	Shoot length (±S.E.)* (cm)	Root length (±S.E.)* (cm)	Shoot length (±S.E.)* (cm)	Root length (±S.E.)* (cm)
	3 dpi^¥^	3 dpi^¥^	5 dpi^¥^	5 dpi^¥^	7 dpi^¥^	7 dpi^¥^
Control	0.56a (±0.03)	4.17a (±0.46)	1.26a (±0.14)	4.85a (±0.22)	2.75a (±0.09)	9.44a (±0.83)
PEPV40	0.92b (±0.10)	4.76a (±0.50)	2.68b (±0.16)	9.29b (±0.22)	3.60b (±0.11)	11.48b (±0.22)

Values followed by the same letter in each treatment are not significantly different from each other at *P* = 0.05 according to Fisher’s Protected LSD (Least Significant Differences). S.E. = Standard Error. *Results from 12 plants per treatment. ^¥^dpi: days post inoculation.

### Spinach growth promotion

**Figure 4:**
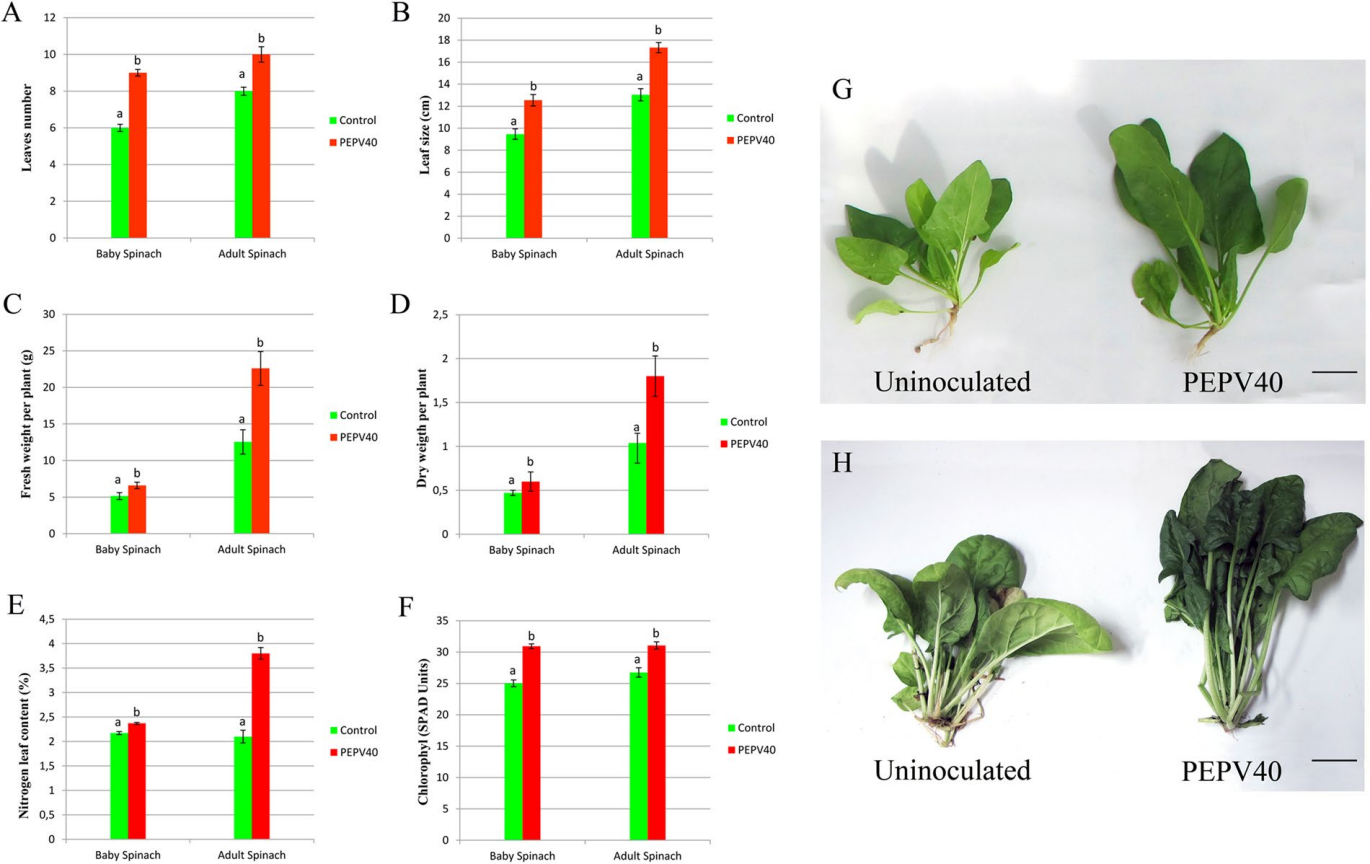
Spinach growth promotion in greenhouse experiments. Number of leaves (**A**) and size (**B**), fresh (**C**) and dry (**D**) weight and nitrogen (**E**) and chlorophyll (**F**) contents. Bars indicate the standard error. Histogram bars marked with the same letter in each treatment are not significantly different from each other at P = 0.05 according to Fisher’s Protected LSD (Least Significant Differences). Baby spinach (**G**, bar 270 mm) and adult spinach (**H**, bar 270 mm) from microcosm experiment.

The results of the greenhouse experiments (Fig. 4, Table 2) showed that PEPV40 promotes the growth of spinach plants, as inoculation with this strain led to an increase in several parameters related to plant growth. Spinach plants have certain peculiarities with regard to their consumption, since the leaves are harvested at different vegetative stages. Baby spinach is consumed raw in salads and more mature spinach plants (large-leafed) are usually cooked before being consumed, either alone or as an ingredient in other dishes. Therefore, the effects of *Rhizobium* inoculation on spinach harvested at these two growth stages was evaluated, where the yield and quality of the leaves were measured. The results of the analysis of these vegetative parameters showed that the plants inoculated with PEPV40 had a significantly higher number of leaves, as well as a larger leaf size and dry and fresh shoot weights (Table 2). The increase in the number and size of leaves ranged from 32.6 to 50% in the inoculated plants, and the increase observed in fresh and dry weights was over 27% in the case of baby spinach and over 73% in the case of mature spinach. Furthermore, plants inoculated with PEPV40 had significantly higher chlorophyll and nitrogen contents, with increases over 13% and 9.2–80.9%, respectively (Table 2). These results clearly show that inoculation with *R. laguerreae* PEPV40 yields bigger spinach plants of higher quality.

**Table 2:** Effect of Rhizobium *laguerreae* inoculation on the growth, nutrient concentrations and chlorophyll content of spinach.

Treatment	Number of leaves (±S.E.)*	Leaf size (cm) (±S.E.)*	SFW (g) (±S.E.)*	SDW (g) (±S.E.)*	Chlorophyll (SPAD units) (±S.E.)*	N (%) (±S.E.)*	P (%) (±S.E.)*	K (%) (±S.E.)*	Mg (%) (±S.E.)*	Ca (%) (±S.E.)*
**Baby spinach**										
Control	6.00a (±0.20)	9.46 a (±0.47)	5.15a (±0.47)	0.47a (±0.03)	25.01a (±0.54)	2.17a (±0.03)	1.24a (±0.03)	7.67a (±0.72)	1.21a (±0.13)	1.30a (±0.02)
PEPV40	9.00b (±0.18)	12.54 b (±0.52)	6.60b (±0.44)	0.60b (±0.03)	30.92b (±0.39)	2.37b (±0.02)	1.18a (±0.03)	7.45a (±0.52)	1.54a (±0.17)	1.34a (±0.03)
**Adult spinach**										
Control	8.00a (±0.22)	13.04 a (±0.55)	12.54a (±1.66)	1.04a (±0.11)	26.75a (±0.75)	2.10a (±0.13)	1.56a (±0.35)	9.61a (±0.89)	1.15a (±0.12)	1.27a (±0.14)
PEPV40	10.00b (±0.42)	17.32 b (±0.47)	22.59b (±2.31)	1.80b (±0.23)	31.04b (±0.59)	3.80b (±0.12)	1.56a (±0.08)	9.89a (±0.43)	1.20a (±0.11)	1.55a (±0.32)

Values followed by the same letter in each treatment are not significantly different from each other at *P* = 0.05 according to Fisher’s Protected LSD (Least Significant Differences). S.E. = Standard Error. SFW = Shoot Fresh Weight. SDW = Shoot Dry Weight. *Results from 12 plants per treatment.

## Discussion

The current concern for a healthy diet and environmental protection has led to the proposal of the use of biological alternatives instead of chemicals in the fertilization of plants. One of the most attractive ways to achieve this goal is the use of plant probiotic bacteria that promote plant growth. Nevertheless, and particularly when plants are consumed raw, the bacteria used in biofertilization must be safe for humans, as discussed by García-Fraile *et al*.^13^. With respect to plant probiotic potential, rhizobia stand out, since these bacteria have been shown to be harmless to human health and the environment after decades of use for legume inoculation^13,14^. Moreover, these bacteria promote the plant growth of non-legumes. *Rhizobium* is a particularly good PGPR for vegetables consumed raw, such as peppers, tomatoes, and lettuce, as we have recently shown^13,20^. Nevertheless, the number and variety of bacterial strains and plants analysed are still few. This is the case of spinach, selected in this study because it is increasingly being consumed raw in salads, but where the plant promotion following inoculation with bacteria has scarcely been analysed. In the work by Çakmakçı *et al*.^37^, increases in shoot weight have been found after the inoculation of strains from the genera *Bacillus*, *Paenibacillus*, and *Pseudomonas*. In the case of rhizobia, it has already been shown that a *Rhizobium* strain can colonize spinach roots, but no significant effects were found in the growth of spinach seedlings^44^. These results render it necessary to extend the studies to other *Rhizobium* strains, because different species of the same genus, or even different strains of the same species, may have different *in vitro* and *in vivo* plant growth promotion abilities. Accordingly, and considering the importance of probiotic bacteria that promote the growth of spinach, we have analysed a strain isolated from *P. vulgaris* nodules, which was identified as *R. laguerreae* after the analysis of *recA* and *atpD* genes, two robust markers for rhizobial taxonomy^45^ which allow the identification of species nodulating *P. vulgaris* ^46^. The species *R. laguerreae* was first isolated from nodules of *Vicia* ^41^, but is known to nodulate other legumes, such as *Trifolium* and *Phaseolus*, in different countries in Africa, Europe, and Asia (Fig. 1). Nevertheless, the plant growth promotion ability of strains from *R. laguerreae* which can be considered suitable for its worldwide use as a biofertilizers, has not been showed to date.

The strain *R. laguerreae* PEPV40 has the basic characteristics of a potentially good PGB because it produces biofilms on abiotic surfaces and cellulose, which are characteristics presented by *Rhizobium* strains that colonize legume and non-legume roots^9,13,20,43,44^. The results of plant colonization assays have confirmed that PEPV40 colonizes spinach roots, forming the typical microcolonies observed in other *Rhizobium* strains that colonize the roots of non-legume plants^9,13,20,21,44^.

Bacterial root colonization is an important step required for promoting plant growth^8,47^. In addition, the production of several metabolites involved in plant growth, such as indole acetic acid and siderophores, and the ability to mobilize plant nutrients, such as nitrogen or phosphorous, are relevant characteristics in bacteria promoting plant growth^13,20^. Among these *in vitro* plant growth promotion mechanisms, the production of IAA and siderophores are the ones most commonly found for *Rhizobium* strains, although some solubilize moderate amounts of phosphate^9^. Although to date most of studies carried out in *Rhizobium* measure IAA by colorimetric-based methods^48^, in this work IAA was also measured by HPLC. The results using this technique detected lower amounts of the hormone, which is a finding that is in agreement with other studies that compare both methods^49,50^. Using the method from Khalid *et al*.^48^, the strain PEPV40 produces IAA in comparable amounts (56.1 mg l^−1^) to those produced by other *Rhizobium* strains that promote the plant growth of non-legumes^13,20^, but also produces higher amounts of siderophores than any of the other strains^13,20^. Therefore, the strain PEPV40 could be a plant growth promoting bacterium, but this ability needs to be confirmed in plant assays.

Both the plant assays carried out *in vitro* and *in vivo* showed that *R. laguerreae* PEPV40 promotes the plant growth of spinach plants. In the initial stages of growth, the spinach seedlings inoculated with the strain PEPV40 have longer shoots and roots than the uninoculated controls, suggesting that this strain exerts a positive effect on plant development, which was confirmed in the microcosm experiments. In both, the young and adult growth stages, the PEPV40 inoculation promotes the growth of the edible parts (shoots) of spinach plants. These plants have a significantly higher number of leaves, which are bigger and have more chlorophyll content, increasing the greenness of the leaves, which is a highly appreciated feature in vegetables with edible leaves. The fresh and dry weights of shoots were also higher in inoculated plants than in uninoculated ones, and contained higher amounts of nitrogen. This effect has already been observed in tomatoes, lettuce, and carrots inoculated with other *Rhizobium* strains^13,20^. This is, however, not due to a fixation of atmospheric nitrogen, as *Rhizobium* is not a free-living nitrogen fixer. Collectively, these results suggest that rhizobia, which increase the N content of legumes via symbiotic nitrogen fixation, can also increase the N content of non-legumes through the promotion of plant growth, which enhances the uptake of soil nutrients.

The results reported here increase the number of *Rhizobium* species that promote plant growth, as well as the number of vegetables suitable for biofertilization with rhizobia. Spinach is consumed fresh throughout the world as an ingredient in salads, including those sold ready-to-eat, and cooked alone or in other dishes of diverse culinary preparations. Additionally, eating spinach is a common practice in many cultures and peoples, and in numerous diets, of which some are ancestral, such as the Mediterranean diet, whose benefits to human health have been proven in recent studies^50,51^. The substitution of the use of chemicals with bacteria, safe to both humans and the environment, in the fertilization of plants that form part of a healthy diet, and at the same time enhance their quality and goodness, is quite an attractive alternative. The safety of rhizobia, backed by several decades of legume inoculation, make these bacteria optimal for the biofertilization of vegetables that are consumed raw worldwide.

## Material and Methods

### Phylogenetic classification

In a previous work, the strain PEPV40 was isolated from an effective nodule of *Phaseolus vulgaris* ^52^. Its classification at species level was performed through the analysis of the concatenated *atpD* and *recA* genes, which were amplified and sequenced as previously described^53^. These sequences were aligned with those of other species from the genus *Rhizobium* using ClustalW software^45^. The distances were calculated according to Kimura’s two-parameter model^54^. A phylogenetic tree was inferred using the neighbour-joining analysis^55^ with MEGA6 software^56^.

### *In vitro* PGP traits

The solubilisation of insoluble phosphate was analysed on plates containing Pikovskaya’s medium^57^, YED-P medium^18^ and NBRIP medium^58^ and adding 0.2% calcium phosphate (CaHPO_4_), tricalcium phosphate and hydroxyapatite (Ca_5_(OH)(PO_4_)_3_), respectively, as P source. The inoculated plates were incubated for 15 days at 28 °C, and then examined for the presence of clear halos surrounding the colonies due to phosphate solubilisation.

Siderophore production was evaluated in M9-CAS-AGAR^59^ modified with the addition of a cationic solvent (HDTMA) to stabilize the Fe-CAS complex, providing the characteristic color^60^. It was also analysed according to Pérez-Miranda *et al*.^42^. The strain PEPV40 was inoculated on plates containing YMA medium^61^, and incubated for five days at 28 °C. Each plate was subsequently overlaid with 12 ml O-CAS medium (60.5 mg l^−1^ chrome azurol S, 72.9 mg l^−1^ hexadecyltrimethylammonium bromide (HDTMA), 30.24 g l^−1^ piperazine-1,4-bis(2-ethanesulfonic acid) (PIPES), 10 ml l^−1^ of a solution containing 1 mM FeCl_3_.6H_2_O in 10 mM HCl and 9 g l^−1^ agarose). After 20 minutes at the most, the surrounding bacterial colonies will have changed colour. The experiment was repeated at least three times.

Indole acetic acid production was evaluated in JMM medium^62^ supplemented with 0.17 g l^−1^ of tryptophan. After five days of incubation, the supernatants were recovered by centrifugation at 5000 × g, and filtered using 0.22 µm Amicon® Millipore filters (Millipore Co., USA). One millilitre of Salkowski agent was then added to 2 ml of supernatant, and the red colour formed was measured by spectrophotometry at 550 nm using an ATI Unicam 8625 Spectrometer (Mattson, USA)^48^. In addition, the IAA was detected by HPLC^63^ at the Elemental Analysis, Chromatography and Mass Spectrometry Service of NUCLEUS (University of Salamanca, Spain), using an Agilent 1100 HPLC and a Trap XCT ion trap mass spectrometer as MS/MS detector. The ascent was expressed using a C18 column that was 10 cm × 2.1 mm in length and internal diameter, respectively, with a 2.7 microparticle using two eluents. Eluent A was water with 0.1% formic acid, and eluent B was water with acetonitrile. The initial conditions were 90% A and 10% B. A gradient was used to reach 20% of B at 3 minutes, and 90% of B at 20 minutes. An IAA D2 internal standard was used in the analysis.

### Cellulose production

Cellulose production was determined on YMA liquid and solid media supplemented with 0.25% Congo Red, which links β 1–4 bonds (present in cellulose-like polysaccharides), and allows the staining of red bacterial colonies^43^. The strains were inoculated in 30 ml of YMB liquid medium^61^, and incubated in an orbital shaker at 28 °C for five days at 180 rpm, followed by static growth for two days at the same temperature. Five millilitres were then recovered from the bottom of the flasks and centrifuged at 1500 × g for 5 min. The flocs were then washed with 5 ml of 100 mM PCA buffer pH 5, recovered by centrifugation, and resuspended in 5 ml of the same buffer. The suspensions were placed on 5 mm diameter petri plates and treated with 10 U ml^−1^ cellulase from *Trichoderma viride* (Sigma Co., USA) for 2 h at 37 °C in an orbital shaker at 180 rpm. Controls without cellulase were incubated under the same conditions.

### Biofilm formation assays

The ability of strain PEPV40 to form biofilms on abiotic surfaces was tested at macroscale following the protocol described by Wang *et al*.^64^ with several modifications. Borosilicate glass tubes were filled with 5 ml of TY medium containing 100 µl of saturated overnight cultures as inocula. After five days of incubation at 28 °C, when the stationary phase had been reached, the content of each tube was gently removed. The tubes were rinsed with water and stained with 6 ml of 0.3% crystal violet for 5 min, and then washed with sterile water, dried and photographed. This ability was also tested at microscale, as described by O’Toole^65^ and modified by Fujishige *et al*.^66^. Briefly, a pre-inoculum of each strain was grown in YMB liquid medium for two days until the culture reached 2.0 OD measured at 600 nm. Bacterial attachment to PVC multi-well plates was assayed by pipetting 100 μl of this normalized suspension into each well. The plates were incubated statically at 28 °C, and revealed with 0.3% crystal violet at specific times (24, 48 and 72 h). The plate wells were washed, and the remaining dye was solubilized with 80% ethanol-20% acetone. Absorbance at 570 nm was measured in an ASYS UVM340 Microtiter Plate Reader (Biochrom, UK), and subsequently analysed.

### GFP-labelling of strains

The GFP-labelling of strains was performed as previously described for rhizobium strains^12^. Briefly, the plasmid pHC60^67^ was introduced into the strains by biparental mating using *E. coli* S17.1^68^ as donor strain. For this mating, fresh cultures of donor and recipient strains were mixed on YMA plates^61^, and then incubated overnight at 28 °C. Transconjugants were selected on minimal medium plates^69^ supplemented with antibiotic (tetracycline at 10 µg/ml). The transfer of pHC60 to strain PEPV15 afforded bacteria expressing the expected GFP, as detected by fluorescence microscopy using a NIKON eclipse 8Oi fluorescence microscope. The recombinant strains were routinely grown at 28 °C in TY medium^70^ supplemented with tetracycline (10 µg/ml).

### Plant colonization and *in vitro* effect on plant growth

Seeds of spinach *(Spinacia oleracea L.)* were surfaced-sterilized with 70% ethanol for 30 s, followed by soaking in an aqueous 5% sodium hypochlorite solution for 5 min. The seeds were then washed five times with sterile water and germinated on water-agar plates. The seedlings were maintained in the dark for three days, and then transferred to 1% agar square plates (12 × 12 cm), with a distribution of five seedlings per plate. The plants were divided into two lots of fifteen for inoculation with the unlabelled strain PEPV40 and with GFP-labelled PEPV40 for testing the effect on early plant growth and for colonization assays, respectively. To prepare the suspensions, the strains were grown for five days at 28 °C on YMA plates flooded with sterile water in order to obtain bacterial suspensions, which were transferred to sterile flasks. The suspensions were adjusted to an OD (600 nm) of 0.5, which corresponds to a final concentration of 10^8^ CFU/ml after counting the number of viable cells using the serial decimal dilution method. A micropipette was used to inoculate each seedling with 250 μl of this suspension at the intersection between the roots and the cotyledons. Fifteen uninoculated spinach seedlings were included as negative control. They were maintained in a growth chamber and observed at three, five and seven days post-inoculation. For colonization assays, and to remove unbound bacteria, the roots were gently washed three times with sterile distilled water before microscopic observation. Fluorescence microscopy was carried out with a Nikon Eclipse 80i, and a mercury lamp was used to for green fluorescent protein excitation. Plant colonization was observed at three, five and seven days post-inoculation. At these times, the lengths of the shoots and the roots of seedlings inoculated with the unlabelled strain were measured.

### Growth promotion assays in microcosm experiments

The ability to promote plant growth was evaluated on *Spinacia oleracea* L. var “Viking” plants using a mix of non-sterilized soil and vermiculite “SEED PRO 6040”/vermiculite (3:1 V/V) (PROJAR, Spain) as substrate. Volumes of 4.8 l of substrate were placed in plastic pots with 5 l capacity. The seeds were directly germinated in this substrate and inoculated with 5 mL of the strain suspensions with a final concentration of 10^8^ CFU mL^−1^ (0.5 OD measured at 600 nm). To obtain these suspensions, the cells of the strain PEPV40 cultivated on YMA plates for five days at 28 °C were suspended in sterile water. Uninoculated spinach plants were included as negative controls under the same conditions. No chemical fertilizers were added. The plants were irrigated with water from a bottom reservoir every 48 h. The plants were maintained in a greenhouse illuminated with natural light in summer (night temperature ranging from 15 to 20 °C, and day temperatures ranging from 25 to 35 °C), with humidity control. From a total of 24 plants per treatment, lots of 12 plants were harvested at two different vegetative stages: baby and adult (regular) spinaches. The number of leaves per plant was counted, and the fresh and dry weights per plant were recorded. Chlorophyll relative content was obtained by the successive measuring of leaves with a chlorophyll meter SPAD-502PLUS (Konica Minolta, Osaka, Japan), avoiding leaves located close to the substrate^71^. The dry plants were used for the analyses of N, P, K, Ca and Mg, which were performed by the Ionomics Service at CEBAS-CSIC (Spain), using Elemental Analyst model TruSpec CN628 equipment for the N analysis, and ICP THERMO ICAP 6500DUO equipment for the analysis of the remaining elements. Statistical analyses were carried out using the StatView program for Macintosh computers. Data were analysed by one-way analysis of variance, and mean values were compared with Fisher’s Protected Least Significant Differences (LSD) test (P ≤ 0.05).

## References

[1] Saini RK, Ko EY, Keum YS (2016). Minimally processed ready-to-eat baby-leaf vegetables: Production, processing, storage, microbial safety, and nutritional potential. Food Rev. Intern..

[2] Castro-Ibáñez I, Gil MI, Allende A (2016). Ready-to-eat vegetables: Current problems and potential solutions to reduce microbial risk in the production chain. Food Sci. Technol..

[3] Wadamori Y, Gooneratne R, Hussain MA (2016). Outbreaks and factors influencing microbiological contamination of fresh produce. J. Sci. Food Agric..

[4] Oyinlola LA, Obadina AO, Omemu AM, Oyewole OB (2016). Prevention of microbial hazard on fresh-cut lettuce through adoption of food safety and hygienic practices by lettuce farmers. Food Sci. Nutr..

[5] Bhardwaj D, Ansari MW, Sahoo RK, Tuteja N (2014). Biofertilizers function as key player in sustainable agriculture by improving soil fertility, plant tolerance and crop productivity. Microb. Cell Fact..

[6] Mahanty T (2016). Biofertilizers: a potential approach for sustainable agriculture development. Environ. Sci. Pollut. Res. Int..

[7] Berlec A (2012). Novel techniques and findings in the study of plant microbiota: Search for plant probiotics. Plant Sci..

[8] Compant S, Clément C, Sessitsch A (2010). Plant growth-promoting bacteria in the rhizo- and endosphere of plants: Their role, colonization, mechanisms involved and prospects for utilization. Soil Biol. Biochem..

[9] Flores-Félix JD (2016). *Rhizobium* as plant probiotic for strawberry production under microcosm *conditions*. Symbiosis..

[10] Pii Y (2015). Microbial interactions in the rhizosphere: beneficial influences of plant growth-promoting rhizobacteria on nutrient acquisition process. A review. Biol. Fert. Soils..

[11] Berg G, Eberl L, Hartmann A (2005). The rhizosphere as a reservoir for opportunistic human pathogenic bacteria. Environ. Microbiol..

[12] Mendes R, Garbeva P, Raaijmakers JM (2013). The rhizosphere microbiome: significance of plant beneficial, plant pathogenic, and human pathogenic microorganisms. FEMS Microbiol. Rev..

[13] García-Fraile P (2012). *Rhizobium* promotes non-legumes growth and quality in several production steps: Towards a biofertilization of edible raw vegetables healthy for humans. PLoS ONE..

[14] Olivares J, Bedmar EJ, Sanjuán J (2013). Biological Nitrogen Fixation in the Context of Global Change. Mol. Plant Microbe. Interact..

[15] Chabot R, Antoun H, Cescas MP (1996). Growth promotion of maize and lettuce by phosphate-solubilizing *Rhizobium leguminosarum* biovar phaseoli. Plant Soil..

[16] Noel TC, Sheng C, Yost CK, Pharis RP, Hynes MF (1996). *Rhizobium leguminosarum* as a plant growth-promoting rhizobacterium: direct growth promotion of canola and lettuce. Can. J. Microbiol..

[17] Antoun H, Beauchamp CJ, Goussard N, Chabot R, Lalande R (1998). Potential of *Rhizobium* and *Bradyrhizobium* species as growth promoting rhizobacteria on non-legumes: effect on radishes (*Raphanus sativus* L.). Plant Soil..

[18] Peix A (2001). Growth promotion of chickpea and barley by a phosphate solubilizing strain of *Mesorhizobium mediterraneum* under growth chamber conditions. Soil Biol. Biochem..

[19] Yanni YG (2001). The beneficial plant growth-promoting association of *Rhizobium leguminosarum* bv. trifolii with rice roots. Australian J. Plant. Physiol..

[20] Flores-Félix JD (2013). Use of *Rhizobium leguminosarum* as a potential biofertilizer for *Lactuca sativa* and *Daucus carota* crops. J. Plant Nutr. Soil Sci..

[21] Granada CE (2014). Diversity of native rhizobia isolated in south Brazil and their growth promotion effect on white clover (*Trifolium repens*) and rice (*Oryza sativa*) plants. Biol. Fert. Soils..

[22] Karthik C, Oves M, Sathya K, Ramkumar VS, Arulselvi PI (2017). Isolation and characterization of multi-potential *Rhizobium* strain ND2 and its plant growth-promoting activities under Cr(VI) stress. Arch. Agron. Soil Sci..

[23] Höflich G, Wiehe W, Kühn G (1994). Plant growth stimulation by inoculation with symbiotic and associative rhizosphere microorganisms. Experientia..

[24] Alami Y, Achouak W, Marol C, Heulin T (2000). Rhizosphere soil aggregation and plant growth promotion of sunflowers by an exopolysaccharide-producing *Rhizobium* sp. strain isolated from sunflower roots. Appl. Environ. Microbiol..

[25] Gutiérrez-Zamora ML, Martínez-Romero E (2001). Natural endophytic association between *Rhizobium etli* and maize (*Zea mays* L.). J. Biotechnol..

[26] Mishra RP, Singh RK, Jaiswal HK, Kumar V, Maurya S (2006). *Rhizobium*-mediated induction of phenolics and plant growth promotion in rice (*Oryza sativa* L.). Curr Microbiol..

[27] Schwedhelm C, Boeing H, Hoffmann G, Aleksandrova K, Schwingshackl L (2016). Effect of diet on mortality and cancer recurrence among cancer survivors: a systematic review and meta-analysis of cohort studies. Nutr, Rev..

[28] Ndanuko RN, Tapsell LC, Charlton KE, Neale EP, Batterham MJ (2016). Dietary Patterns and Blood Pressure in Adults: A Systematic Review and Meta-Analysis of Randomized Controlled Trials. Adv. Nutr..

[29] Medina-Remón A, Kirwan R, Lamuela-Raventós RM, Estruch R (2016). Dietary Patterns and the Risk of Obesity, Type 2 Diabetes Mellitus, Cardiovascular Diseases, Asthma, and Mental Health Problems. Crit. Rev. Food Sci. Nutr..

[30] Martínez-González MA, Martín-Calvo N (2016). Mediterranean diet and life expectancy; beyond olive oil, fruits, and vegetables. Curr. Opin. Clin. Nutr. Metab. Care..

[31] Schwingshackl L, Hoffmann G (2016). Does a Mediterranean-Type Diet Reduce Cancer Risk?. Curr. Nutr. Rep..

[32] Hariharan D, Vellanki K, Kramer H (2015). The Western Diet and Chronic Kidney Disease. Curr. Hypertens Rep..

[33] Di Daniele N (2017). Impact of Mediterranean diet on metabolic syndrome, cancer and longevity. Oncotarget..

[34] Petersson SD, Philippou E (2016). Mediterranean diet, cognitive function, and dementia: A systematic review of the evidence. Adv. Nutr..

[35] Yoon YE (2017). Influence of cold stress on contents of soluble sugars, vitamin C and free amino acids including gamma-aminobutyric acid (GABA) in spinach (*Spinacia oleracea*). Food Chem..

[36] FAOSTAT. Crops data for 2011. Food and Agricultural Organization of the United Nations. http://faostat3.fao.org (2013).

[37] Çakmakçı R, Erat M, Erdoğan Ü, Dönmez MF (2007). The influence of plant growth–promoting rhizobacteria on growth and enzyme activities in wheat and spinach plants. J. Plant Nutr. Soil Sci..

[38] Thilakarathna MS, Raizada MN (2017). A meta-analysis of the effectiveness of diverse rhizobia inoculants on soybean traits under field conditions. Soil Biol. Biochem..

[39] Bashan Y, de-Bashan LE, Prabhu SR, Hernández JP (2014). Advances in plant growth-promoting bacterial inoculant technology: formulations and practical perspectives (1998–2013). Plant Soil..

[40] Kuykendall, L. D., Young, J. M., Martínez-Romero, E., Kerr, A. & Sawada, H. *Rhizobium* in *Bergey’s Manual of Systematics ofArchaea and Bacteria* (ed. John Wiley & Sons.) 1–36 (2015).

[41] Saïdi S (2014). *Rhizobium laguerreae* sp. nov. nodulates *Vicia faba* on several continents. Int. J. Syst. Evol. Microbiol..

[42] Pérez-Miranda S, Cabirol N, George-Téllez R, Zamudio-Rivera LS, Fernández FJ (2007). O-CAS, a fast and universal method for siderophore detection. J. Microbiol. Meth..

[43] Robledo M (2012). Role of *Rhizobium* endoglucanase CelC2 in cellulose biosynthesis and biofilm formation on plant roots and abiotic surfaces. Microb Cell Fact..

[44] Jiménez-Gómez, A. *et al*. Effective Colonization of Spinach Root Surface by *Rhizobium* in *Biological Nitrogen Fixation and Beneficial Plant-Microbe Interaction*. (ed. González-Andrés, A. & James, E.) 109–122 (Germany, 2016).

[45] Zhang YM, Tian CF, Sui XH, Chen WF, Chen WX (2012). Robust markers reflecting phylogeny and taxonomy of rhizobia. PLoS One..

[46] Rouhrazi, K., Khodakaramian, G., & Velázquez, E. Phylogenetic diversity of rhizobial species and symbiovars nodulating *Phaseolus vulgaris* in Iran. *FEMS microbiol letters*. 363(5) (2016). 10.1093/femsle/fnw024 26832644

[47] Lugtenberg B, Kamilova F (2009). Plant-Growth-Promoting Rhizobacteria. Annu. Rev. Microbiol..

[48] Khalid A, Arshad M, Zahir ZA (2004). Screening plant growth-promoting rhizobacteria for improving growth and yield of wheat. J. Appl. Microbiol..

[49] Crozier A, Arruda P, Jasmin JM, Monteiro AM, Sandberg G (1988). Analysis of indole-3-acetic acid and related indoles in culture medium from *Azospirillum lipoferum* and *Azospirillum brasilense*. App. Environ. Microb..

[50] Fuentes-Ramírez L, Jiménez-Salgado T, Abarca-Ocampo IR, Caballero-Mellado J (1993). *Acetobacter diazotrophicus*, an indoleacetic acid producing bacterium isolated from sugarcane cultivars of Mexico. Plant Soil..

[51] Dernini S (2016). Med Diet 4.0: the Mediterranean diet with four sustainable benefits. Public Health Nutr..

[52] Flores-Félix J.D., *et al*. MALDI-TOFFMS, a tool for diversity analysis and detection of novel rhizobial species nodulating *Phaseolus vulgari*s. Microorganisms for future agriculture. Spain: Spanish Society of Nitrogen Fixation (SEFIN), Latin American Society Rhizobiology (ALAR) and University of Sevilla. 25–26 (2013).

[53] Gaunt MW, Turner SL, Rigottier-Gois L, Lloyd-Macgilp SA, Young JWP (2001). Phylogenies of *atpD* and *recA* support the small subunit rRNA-based classification of rhizobia. Int. J. Syst. Evol. Microbiol..

[54] Kimura M (1980). A simple method for estimating evolutionary rates of base substitutions through comparative studies of nucleotide sequences. J. Mol. Evol..

[55] Saitou N, Nei M (1987). A neighbour-joining method: a new method for reconstructing phylogenetics trees. Mol Biol Evol..

[56] Tamura K (2011). MEGA5: molecular evolutionary genetics analysis using maximum likelihood, evolutionary distance, and maximum parsimony methods. Mol Biol Evol..

[57] Pikovskaya RI (1948). Mobilization of phosphorus in soil connection with the vital activity of some microbial species. Microbiologiya..

[58] Nautiyal CS (1999). An efficient microbiological growth medium for screening phosphate solubilizing microorganisms. FEMS Microbial Lett..

[59] Schwyn B, Neilands JB (1987). Universal chemical assay for the detection and determination of siderophores. Anal Biochem..

[60] Alexander DB, Zuberer DA (1991). Use of Chrome Azurol S reagents to evaluate siderophore production by rhizosphere bacteria. Biol. Fertil. Soils..

[61] Vincent, J. M. The cultivation, isolation and maintenance of rhizobia in *A Manual for the Practical Study of* Root-Nodule (ed. Vicent, J. M.) 1–13 (Oxford, 1970).

[62] O’Hara GW, Goss TJ, Dilworth MJ, Glenn AR (1989). Maintenance of intracellular pH and acid tolerance in Rhizobium meliloti. Appl. Environ Microbiol..

[63] Diez-Méndez A, Rivas R (2017). Improvement of saffron production using *Curtobacterium herbarum* as a bioinoculant under greenhouse conditions. Aims Microbiol..

[64] Wang H, Zhong Z, Cai T, Li S, Zhu J (2004). Heterologous overexpression of quorum-sensing regulators to study cell-density-dependent phenotypes in a symbiotic plant bacterium Mesorhizobium huakuii. Arch. Microbiol..

[65] O’Toole GA (1999). Genetic approaches to study of biofilms. Method enzymol..

[66] Fujishige NA, Kapadia NN, De Hoff PL, Hirsch A (2006). Investigations of *Rhizobium* biofilm formation. FEMS Microbiol. Ecol..

[67] Cheng HP, Walker GC (1998). Succinoglycan is required for initiation and elongation of infection threads during nodulation of alfalfa by *Rhizobium meliloti*. J. Bacteriol..

[68] Simon, R., Priefer, U. & Puhler, A. Vector plasmids for *in vivo* and *in vitro* manipulations of gram negative bacteria in *Molecular Genetics of the Bacteria-Plant Interaction* (ed. Puhler, A.) 98–106 (Berlín, 1983).

[69] O’gara F, Shanmugam KT (1976). Control of symbiotic nitrogen fixation in rhizobia. Regulation of NH_4_^+^ assimilation. Biochem Biophys. Acta..

[70] Beringer JE (1974). R factors transfer in *Rhizobium leguminosarum*. J. Gen Microbiol..

[71] Shahzad SM, Arif MS, Riaz M, Iqbal Z, Ashraf M (2013). PGPR with varied ACC-deaminase activity induced different growth and yield response in maize (*Zea mays* L.) under fertilized conditions. Eur. J. Soil Biol..

